# Energy Consumption Optimization of a Fluid Bed Dryer in Pharmaceutical Manufacturing Using EDA (Exploratory Data Analysis)

**DOI:** 10.3390/s23083994

**Published:** 2023-04-14

**Authors:** Roberto Barriga, Miquel Romero, Houcine Hassan, David F. Nettleton

**Affiliations:** 1 Industrias Farmacéuticas Almirall, Ctra. N-II, Km. 593, 08740 Sant Andreu de la Barca, Spain; 2 Departamento de Informática de Sistemas y Computadores, Universitat Politècnica de València, Camino de Vera, No. 14, 46022 Valencia, Spain; 3 IRIS Technology Solutions, Ctra. d’Esplugues, 39, 08940 Cornella de Llobregat, Spain

**Keywords:** energy optimization, smart sensors, industrial process, machine learning, pharmaceutical fluid bed dryer, control and monitoring

## Abstract

In this paper, a data preprocessing methodology, EDA (Exploratory Data Analysis), is used for performing an exploration of the data captured from the sensors of a fluid bed dryer to reduce the energy consumption during the preheating phase. The objective of this process is the extraction of liquids such as water through the injection of dry and hot air. The time taken to dry a pharmaceutical product is typically uniform, independent of the product weight (Kg) or the type of product. However, the time it takes to heat up the equipment before drying can vary depending on different factors, such as the skill level of the person operating the machine. EDA (Exploratory Data Analysis) is a method of evaluating or comprehending sensor data to derive insights and key characteristics. EDA is a critical component of any data science or machine learning process. The exploration and analysis of the sensor data from experimental trials has facilitated the identification of an optimal configuration, with an average reduction in preheating time of one hour. For each processed batch of 150 kg in the fluid bed dryer, this translates into an energy saving of around 18.5 kWh, giving an annual energy saving of over 3.700 kWh.

## 1. Introduction

The economic environment, the constant regulatory measures applied by administrations to maintain low healthcare costs, as well as the changes in healthcare regulations that have been introduced in recent years, have all had a significant impact on the pharmaceutical and healthcare industries. The Industry 4.0 paradigm encompasses changes in the traditional production model of the pharmaceutical industry with the inclusion of technologies that go beyond conventional automation [1]. The ultimate goal is to produce more cost-efficient drugs through the optimal incorporation of technologies such as advanced analytics [2]. It is worth mentioning that the massive amounts of data generated by the industries cannot be exploited in many cases due to the complexity involved. Two key aspects addressed in this paper are (i) the amortization of older fluid bed dryers that do not have the latest sensors and (ii) the complexity of the process to be modeled:

- Firstly, due to the situation described above, and the high cost of the fluid bed dryer involved in the drug production process, it is common practice to try to maximize the useful life of these fluid bed dryers. Thus, in particular, legacy fluid bed dryers (more than 15 years old) are not equipped with the latest sensors.
- Secondly, in the pre-heating process, there is a significant variability of time that depends on when the operator has started and finished the heating process. These operations are executed manually by the operators, as the fluid bed dryer is an old machine, and it is not equipped with sensors that detect when it is the optimal time to end the preheating process. On the one hand, there are operators who keep the fluid bed dryer warming up for approximately 45 min, whereas other operators keep the fluid bed dryer warming for up to 115 min. Although this scenario does not apply to all industrial plants, it might apply to several plants.
- Thirdly, at first glance it seems that the process is simple, and the formula that describes it is, basically, “the outlet temperature minus the inlet temperature” which approaches a constant value. However, in reality, it is a process with a complex non-linear behavior where the required temperature and therefore the time varies depending on different parameters such as the batch, the quantity, the mixture, and so on.

Furthermore, with reference to the third point, the difference between the outlet temperature and the inlet temperature is sufficient to fix the equation for the state of the water in the air as long as we have constant pressure. However, this is not typically the case because the state of the filters varies according to their saturation state [3].

On the other hand, the weight compensation related to the richness of the API (Active Pharmaceutical Ingredients) implies that the mass to be dried is not always the same, and also the size of the agglomerates does not remain constant because it depends on the previous kneading. Thus, there is a non-linearity with an IN-OUT difference that does not remain constant. In the literature, relevant studies can be found regarding the correlation between inlet and outlet temperatures of the drying air, both through mechanistic and machine learning models. In [4], the authors provide examples of how mathematical modeling can be used in various aspects of pharmaceutical development and manufacturing, such as predicting the behavior of drug molecules in different formulations and optimizing manufacturing processes to increase efficiency and reduce costs. There are other experimental results from batch fluidized bed drying of two pharmaceutical powders, where process parameters are varied, such as inlet air temperature, inlet air velocity, and bed height. Then, in [5], the authors use the desirability approach to analyze the performance of the process based on multiple quality criteria, such as the final moisture content, the drying rate, and the product uniformity. Ref. [6] present a dynamic model of the fluidized bed dryer that takes into account the complex interactions between the particle properties, the gas flow, and the drying process. This model is used to design an observer-based soft sensor that estimates the moisture content based on measurements of other process variables, such as temperature and humidity These authors focus on improving the fluidized bed drying process and the challenges associated with controlling the process, particularly with respect to maintaining consistent product quality. Since we are focused in the current work on the preheating process, where no product is being dried, our approach is to reduce the fluidized bed drying process time, and consequently reduce costs related to energy usage and human operator time.

In this paper, we propose a methodology based on data exploration, also known as EDA (Exploratory Data Analysis), applied to the data of the sensors of the FAMP68 fluid bed dryer. The goal of applying this methodology is to optimize the process of preheating by reducing its duration and therefore the overall energy consumption of the fluid bed dryer. The results obtained show that it is possible to reduce the preheating time of the FAMP68 fluid bed dryer by more than 50%. This represents an average reduction of 1 h per processed batch, achieving an energy savings of around 18.5 kWh per batch, giving a potential annual energy savings of about 3700 kWh (200 batches/year × 18.5 kWh per batch).

The remainder of the paper is structured as follows: Section 2 reviews the literature relevant to the methods and techniques used in the paper; Section 3 explains the functionality of the fluid bed dryer and the main issue to be solved (optimize pre-heating time); Section 4 explains the data collection and the pre-processing stage; Section 5 describes the experimental design; next, Section 6 presents the data exploration, including the identification of trends and system behavior, and verification of the hypothesis with historical data from production batches; finally, Section 7 presents the conclusions and summarizes the work.

## 2. Related Work

In this paper, we study how to optimize energy consumption in a fluid bed dryer for pharmaceutical granules that is not equipped with the latest sensor technologies. This is conducted through exploration methodologies and techniques such as pre-processing, data exploration, and visualization [7,8,9,10]. Data exploration and analysis are among the most important and most intensive steps in any experiment where large amounts of data are being used [11]. Typically, the data are presented in non-optimal formats, or the amount of data and variables to analyze is very extensive. Therefore, it is necessary to identify meaningful variables that affect the process, observe their behavior patterns, and find correlations between these relevant variables to be able to draw conclusions that are beneficial to our objective of optimizing the pre-heating time.

Psychrometrics is a key technique used in the paper for modeling the pre-heating and drying process. Psychrometrics is a subscience of physics dealing with the properties and processes typically of moist air (the gas phase of H_2_O), which can be broadened to cover mixtures of the gas of one substance and the condensation vapor of a second substance [12]. The psychrometric chart is a useful and easy to use tool for determining moist air’s psychrometric properties and visualizing the changes of properties in a sequence of psychrometric processes. Example processes would be (a) as the outside and return air mixes; (b) air passing through heating and cooling coils; (c) the supply fan/duct; (d) the conditioned space [12]. In the literature, the psychrometric model has been the basis of research in data exploration and modeling for complex systems. For example, in a meteorological context, ref. [13] developed a new model of the THI (Temperature-Humidity Index) which represented a simplification of the current NWS (National Weather Service) model (3 parameters vs. 16 for the NWS model). In [14], a Monte-Carlo simulation model was developed to predict the drying behavior of lumber in batch kilns. The drying rates were approximated by a novel combination of high and low moisture asymptotic rates, which provide a simple correction procedure to compensate for the temperature and the humidity variations. In [15], an artificial neural network (ANN)-based psychrometric chart was used for real-time calculations of the air properties required in the drying of agricultural and food materials and in the ventilation of farm buildings. In [16], mathematical models were developed of the psychrometric chart. In the latter, the aim was to identify and model dynamic mathematical relationships between psychrometric properties. Theoretical and empirical models were compared, the latter using a two-layer neural network as a transfer function for the relative humidity of the air.

## 3. Fluid Bed Dryer

The fluid bed dryer is a common piece of equipment widely used in the pharmaceutical industry. The material is dried throughout the granulation process to get the optimum moisture content in the tablet formulation granules needed for effective tablet compression. The drying process involves the transmission of hot air and the extraction of the product’s humidity via diffusion and forced convection. The granulate is fluidized by hot air and dehumidified during this process, resulting in a mass and energy conversion [17].

**Figure 1:**
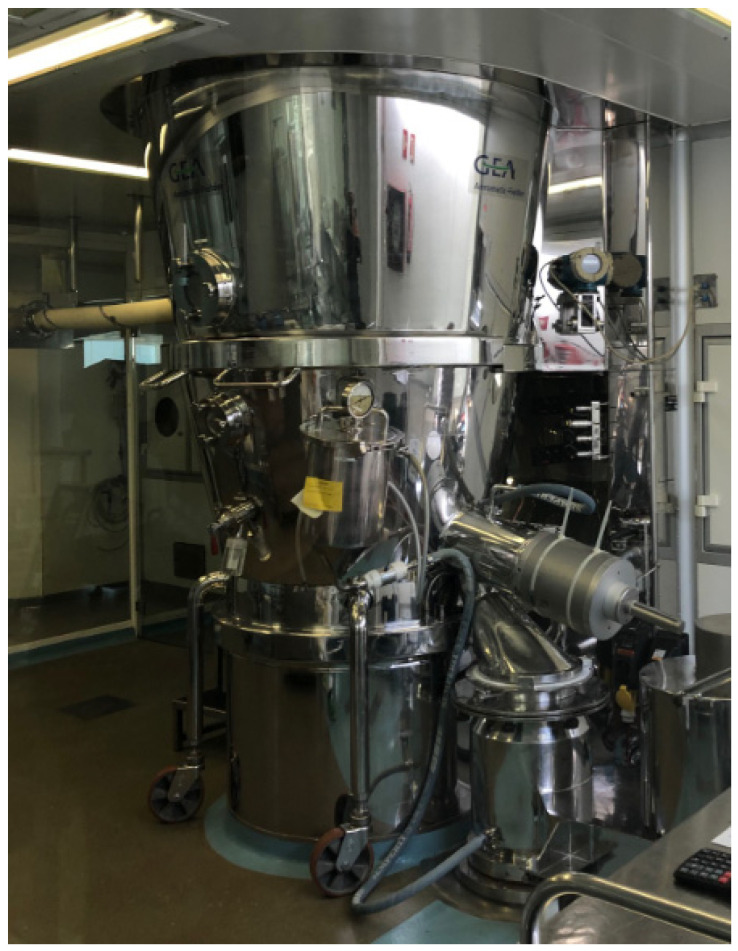
Fluid bed dryer model Fielder Aeromatic MP 6/8.

The fluid bed dryer has three critical parameters that characterize the drying process’s efficiency and thus can influence the final product quality. Temperature, humidity, and air flow are the parameters described in [18]. In theory, a higher temperature and flow rate of the fluid bed dryer’s inlet air imply a shorter drying time. However, depending on the type of product, each of these three parameters must be configured correctly to avoid quality issues and deterioration of the final product obtained after the drying process. It is critical to note that the inlet air temperature should not exceed the critical temperature of the product to be dried so that its quality or pharmaceutical properties are not jeopardized. The fluid bed dryer used in this study was Fielder Aeromatic MP 6/8 (FAMP68), which is shown in Figure 1. This fluid bed dryer has 56 sensors governed by SCADA (Supervisory Control And Data Acquisition), through which the operators monitor and configure the basic parameters of the fluid bed dryer such as the inlet air temperature or the air flow. In the initial phase of the dryer operation, fluid bed dryers are preheated for a certain time, to ensure that the interior of the fluid bed is at the temperature indicated in the formula of the pharmaceutical tablet or pill. Once we have the fluid bed dryer preheated (in a similar fashion to an oven), we introduce the wet granulated product and inject hot air at a constant speed to start the fluidization process, by which the moisture is removed. This process is monitored by the operator through SCADA, which records the increase in outlet air temperature as the product is being dried. Based on the fluid bed drier data sensors, the outlet air and inlet air temperatures are almost the same when the product becomes dry. At this point it is critical to stop the operation, since lengthening it more than necessary could put the quality of the product at risk, as well as consume more time and energy than necessary for the process [19].

The FAMP68 fluid bed dryer used for this experiment is more than 20 years old, and it lacks sensors that indicate when the fluid bed dryer has reached the optimal temperature for the different drying phases (preheating, drying, and cooling), so human operators typically use fixed times for the drying phases. However, for the preheating phase, the time can vary depending on the experience of the operator with the fluid bed dryer. In this paper, we are going to focus on the optimization of the first phase of the process, that is, the pre-heating phase, the previous step before loading any product. Since this process relies on the input of the operator, we suspect that a significant amount of time and energy can be saved using quantitative methods.

## 4. Data Capture and Pre-Processing

**Figure 2:**
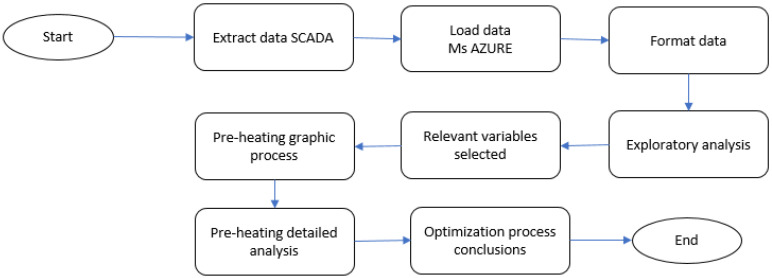
Proposed methodology.

This section will explain how the data capture and pre-processing is carried out. Figure 2 shows the general process that will be followed in Section 4, Section 5 and Section 6 of the paper.

### 4.1. Data Capture

**Figure 3:**
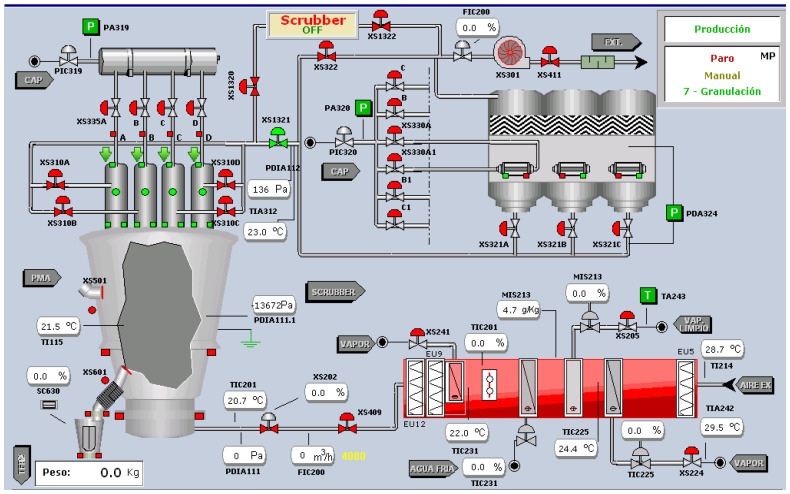
SCADA (Supervisory Control And Data Acquisition).

As shown in Figure 2, the first step is to capture data from the different sources. In our case, we have the FAMP68 fluid bed dryer that is currently operating in a pharmaceutical plant. The FAMP68 fluid bed dryer processes two batches of granules per day, with each batch containing approximately 150 kg of drug mixed with 25 kg of alcohol and 10 kg of another excipient prior to introduction into the fluid bed dryer. The fluid bed dryer has 56 sensors that measure, among other things, inlet/outlet air temperature, air flow in m^3^/h, motor rotation speed, and air pressure. The data will be inside a matrix *D* with *m* columns and *n* rows, where *n* corresponds to the time measured at one minute intervals and *m* to the 56 sensors of the fluid bed dryer. We have two years of data, which equates to over 700,000 readings of each of the 56 signals. A PLC (Programmable Logic Controller, Barcelona, Spain) collects the data and stores them in SCADA (Supervisory Control And Data Acquisition). Figure 3 depicts the SCADA that operators use to interact with the fluid bed dryer (start/stop controller, inlet air temperature indicator, inlet air flow indicator, and so on). The SCADA data was exported to a .csv file format. The table contains over 700.000 rows and 56 columns.

After saving the data in a file, it is uploaded to a cloud computing platform to be processed. Because of the large amount of data (over 3 GB), the Azure platform and its advanced analytics module Databricks using Python were chosen for data analysis.

### 4.2. Data Pre-Processing

Due to the large amount of information, firstly the most relevant variables have to be analyzed and cleaned in order to facilitate the identification of patterns and groupings in the data. To normalize the data, it is observed that the data produced by some sensors (columns) do not vary over time. So, we proceed to eliminate these columns to simplify the data set and to focus on the data from sensors that can help optimize the preheating process of the fluid bed dryer. Overall, we find 10 sensors that do not provide us with any relevant information about the preheating or drying process, as they remain constant over the 2-year period and can therefore be eliminated from the dataset.

## 5. Experiment Design

Firstly, we use psychrometrics to identify the most suitable sensors available to model the behavior of the pre-heating process. The vapor pressure is the outermost curve, which marks the water-vapor change of state. The pressure of change of state increases with increasing temperature, therefore, if we carry out a process with constant humidity, we have that the partial pressure of the water in the mixture increases with increasing temperature. For this reason, the constant RH (Relative Humidity) curves are increasing. In our fluidized bed drying process, we are carrying out a constant pressure process. This causes the air entering the chamber to travel along a horizontal line starting from an initial point at the entrance to the chamber. The movement of the point is going to follow the horizontal line to the left that starts from the initial point because, by absorbing the water from the granules, the humidity of the air is going to increase. The air can continue to absorb water as long as this line does not reach the Dew Point, at which time the air will be saturated with moisture. Fluidized bed dryers are designed so that the inlet air is sufficiently hot and dry so that the Dew Point line is long enough to absorb all the moisture in the granules. According to the psychrometric chart, the psychrometric ratio is defined formally as the ratio of the heat transfer coefficient to the product of mass transfer coefficient and humid heat at a wetted surface. It is quantified using the following equation, where r = psychrometric ratio, $h_{c}$ = convective heat transfer coefficient (Wm^−2^K^−1^), $k_{y}$ = convective mass transfer coefficient (kg m^−2^ s^−1^), and $c_{z}$ = humid heat (J kg^−1^K^−1^).

(1) $$r=\frac{h_{c}}{k_{y}c_{z}}$$

**Figure 4:**
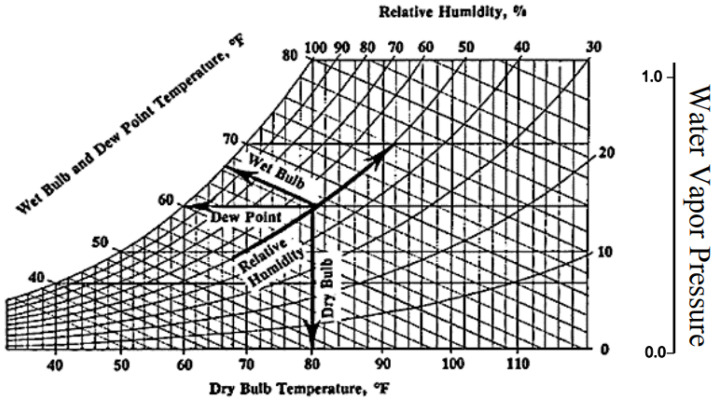
Psychrometric chart.

Taking into account the psychrometric chart in Figure 4 explained above, the following sensors were selected in order to characterize the preheating phase of the fluid bed dryer:

- Fan motor: the signal shows when the fluid bed dryer is on or off.
- Air flow: the signal indicates the air flow (quantity in m^3^/h) that enters the fluid bed dryer. This is configured by the fluid bed dryer operator.
- Inlet air temperature: the signal indicates the temperature at which the air enters the fluid bed dryer and is also set by the operator at the beginning of the process.
- Outlet air temperature: the signal indicates the temperature at which the air exits the fluid bed dryer.

Following the principle of the psychrometric process, once the fluid bed dryer is running and the hot air inlet process begins, we have to take into account the heat retained by the fluid bed dryer to reach preheating temperature. This means that we can rely on the sensor that indicates the temperature of the outlet air of the fluid bed dryer to know how much heat the fluid bed dryer is absorbing. By subtracting the air inlet and outlet temperatures, we can detect when the fluid bed dryer is not capable of absorbing more heat and therefore the inlet air temperature will be similar to the outlet air temperature.

As a last step, the data from the selected sensors will be graphically visualized, on randomly selected days, to observe the behavior of the fluid bed dryer signals when carrying out the preheating, drying, and cooling process each time a batch of pharmaceutical product is processed. To perform this analysis, we will consider the temperature differences of the air inlet and outlet of the fluid bed dryer, as we have commented previously, a variable that we will define as:

(2) $${TA}_{D}={TA}_{s}-{TA}_{e}$$

where ${TA}_{s}$ is the outlet air temperature, ${TA}_{e}$ the inlet air temperature and ${TA}_{D}$ is the temperature difference.

Next, the proposed methodology used in the experiments is summarized. The data will be inside a matrix *D* with *m* columns and *n* rows, where *n* corresponds to the time measured minute by minute and *m* to the 56 sensors of the fluid bed dryer. We define the variables that measure the airflow, fan motor, and phase as Cϕ, Mσ, and F~, respectively. The variables used to select data by days and batches are also defined as QD and QL, respectively. PS will indicate the drying process and *t* the time.

The pseudo code of the data exploration process is the following:

1. Define a matrix D [m, n]
2. Assign data extracted from SCADA to D
3. Upload D in the cloud environment (Azure)
4. Eliminate values with variability equal to zero in D
5. Perform data exploration on D
6. Select key variables D~(${TA}_{e}$ ${TA}_{s}$ CϕMσF~)
7. Select key rows D~ ~ ($Q_{D}$ $Q_{L}$)
8. Perform data exploration on D~ ~ for PS
9. Establish trends over time for t (D~ ~, PS)
10. Establish trends over time for ${TA}_{D}$ (D~ ~, PS)

Hence, the process embodies a typical data analysis/exploration methodology, which will be explained in detail in the next section. It will be seen that visualization of the variables through plots is a key aspect (lines 5 and 8 above), in which the trends over time are established. Also note that previously, using the psychrometric chart (Figure 4), the optimum values of ${TA}_{s}$, ${TA}_{e}$, and ${TA}_{D}$ were established. 

## 6. Data Exploration

The following section describes the data exploration and discovery of trends and systemic behavior. Firstly, in Section 6.1, the data are explored using different samples of days to identify trends and evaluate the behavior of the system (hypothesis); then, in Section 6.2, the hypothesis is verified with all the real production data obtained from the fluid bed dryer for two years.

### 6.1. Data Exploration to Identify Trends and System Behavior

In Table 1, we can observe the information of 4 sensors selected from the total of 56 sensors of the fluid bed dryer, with the selection based on the psychometric air process approach. The information presented in Table 1 corresponds to the number of sensor readings (count), the average value of each sensor (mean), the standard deviation of each sensor (std), as well as the maximum and minimum values, and the limit of each of the quartiles for each sensor. Knowing this preliminary information allows us to establish the average values of the sensors, with respect to individual batches sampled on different days, in order to identify trends and to evaluate the behavior of the system.

**Figure 5:**
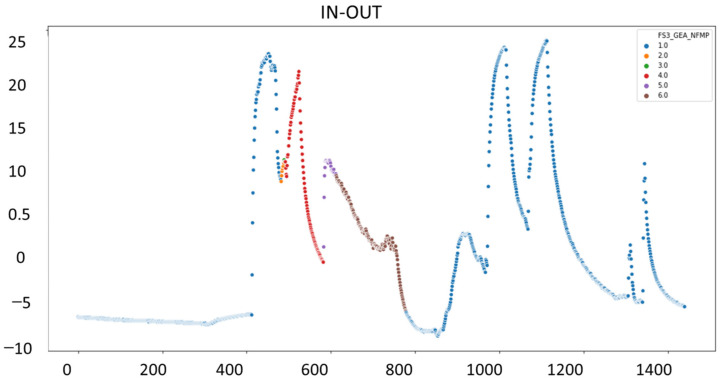
Plot of phases of the drying process.

Figure 5 indicates on the x-axis the elapsed time (1400 min in total) and on the y-axis the difference in temperature of the fluid bed dryer’s inlet and outlet air.

**Figure 6:**
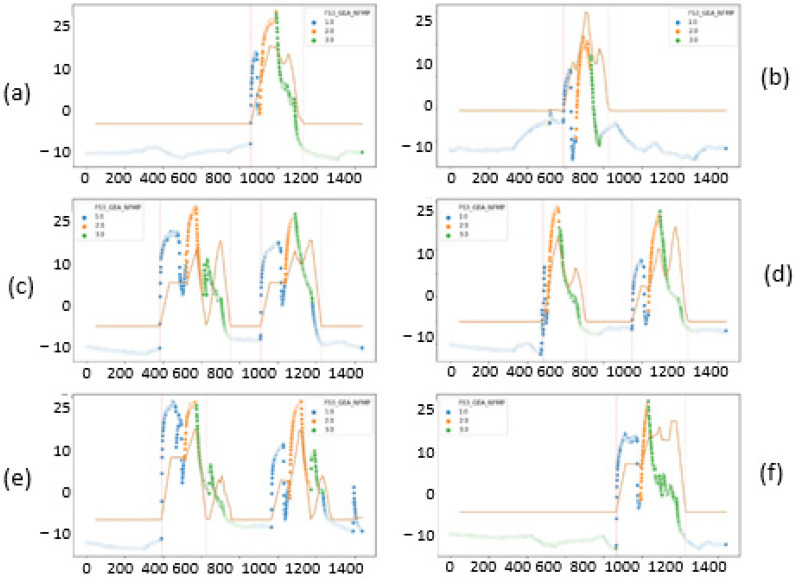
Example of 6 days of batch drying (from left to right and top to bottom): (**a**) 26 January 2018, (**b**) 30 August 2018, (**c**) 7 February 2019, (**d**) 7 February 2019, (**e**) 6 April, and (**f**) 25 January 2018.

To visualize graphically the behavior of the signals on different days, we randomly selected several days on which the fluid bed dryer had been operating to analyze the pattern that follows the preheating, drying, and cooling process. This is illustrated in Figure 6a–f, where each plot indicates a full day’s operation of the fluid bed dryer (in Figure 6a,b,f there was only one batch run during the day, whereas in Figure 6c–e it can be seen there were two consecutive batch runs).

In Table 2, we can see the four most relevant variables that we are going to use for the experiment, selected from the first data exploration performed. We added a new variable (fan motor) that indicates to us when the fluid bed dryer is running. The IF signal (phase indicator) can take the values 1, 2, or 3, depending on the phase the fluid bed dryer is in. Phase 1 corresponds to the preheating phase, where the fluid bed dryer needs to warm up through the hot air inlet to be able to start the drying process (which corresponds to the value 2). When the IF signal (phase indicator) acquires the value 3, it means that the drying process has concluded (phase 2), and therefore the fluid bed dryer must be cooled with air inlet to a lower temperature for the environmental conditioning that will avoid condensation when cooling. The TAE (inlet air temperature) signal corresponds to the degrees to which the air enters the fluid bed dryer for any of the three phases (1: preheat the fluid bed dryer, 2: dry the product, 3: cool the fluid bed dryer). The TAS (outlet air temperature) signal corresponds to the temperature in degrees of the air coming out of the fluid bed dryer. The CAE signal (inlet air flow) indicates the volume of air per unit of time supplied by the fluid bed dryer’s fan, and finally the MOT (fan motor) signal is used to know when the fluid bed dryer is activated in any of the three phases (when the fan motor starts). In Table 2, we can see the different signals, as well as their mean, maximum, and minimum values for a random sample of signals. Note that it is expected to see null values for the minimums of the inlet and outlet temperatures.

**Figure 7:**
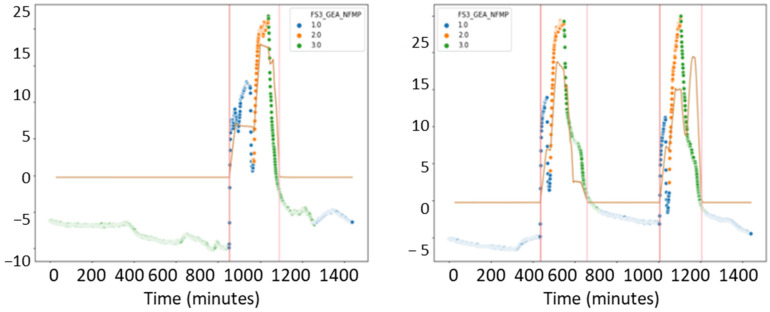
Batches produced per day. One batch (**left**); two batches (**right**).

The first action that has been performed is finding out how many batches of product are dried in the fluid bed dryer each day. To do this, a random sample of signals is taken using only those in which we have the fan motor running (MOT > 1). In Figure 7, the x-axis indicates the number of minutes elapsed in a day (we see that the total is 1400 min, corresponding to 24 h), and the y-axis corresponds to the difference of inlet and outlet temperature (TAE) and output (TAS) of the fluid bed dryer. Each color corresponds to a phase of the IF signal (phase indicator). The value 1 corresponds to the preheating phase (blue), value 2 to the drying phase (orange), and the value 3 to the cooling phase (green) of the fluid bed dryer. We also analyzed how much time (in minutes) it takes on average to complete the three drying phases: preheating, drying, and cooling, as shown in Figure 7. Some days are selected with a single batch, and others selected with two or more batches to observe the behavior of the fluid bed dryer. We note that the fluid bed dryer requires approximately 300 min (or 5 h) to dry a batch of product on average.

Once the average drying time of a batch is known, including its three phases (preheating the fluid bed dryer, drying the product, and cooling the fluid bed dryer), which corresponds to approximately 5 h, we focus on the preheating phase, indicated by the blue dots in Figure 7. The goal is to know how much time it takes to heat up the dryer before starting the drying process. Since we are going to focus on the preheating phase, we will select the data that meet the condition IF = 1 (preheat phase) and CAU > 0 (airflow greater than zero), and we will choose a day to identify the duration in minutes of the preheating phase.

**Figure 8:**
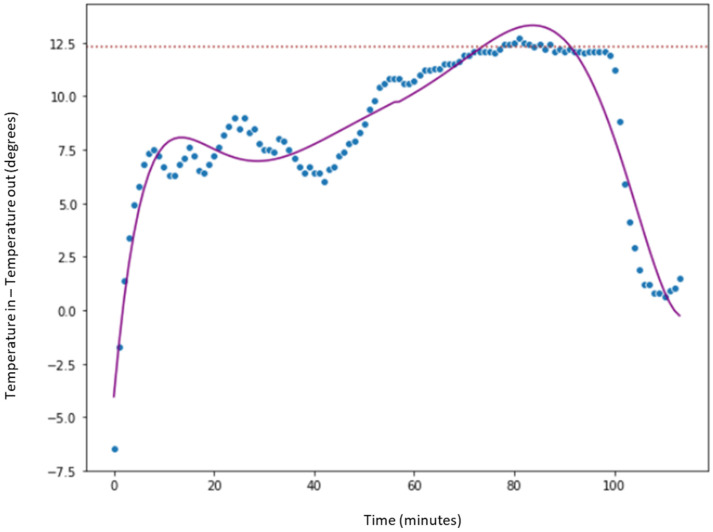
Preheating phase analysis of one batch of product.

In Figure 8, the blue dots represent the difference between the inlet (TAE) and outlet (TAS) temperatures. The horizontal red line at 12.5° indicates the maximum of the values (blue dot). The x-axis corresponds to the time in minutes of the preheating phase IF = 1.

The data consist of a set of points (x_j_, y_j_), j = 1, …, n, where x_j_ is an independent variable and y_j_ is an observed value. The data are processed with a set of m convolution coefficients *C_i_*, expressed mathematically as

(3) $$y_{j}=\sum_{i=\frac{1-m}{2}}^{\frac{m-1}{2}}C_{i}y_{j+i},\frac{m+1}{2}\leq j\leq n-\frac{m-1}{2}$$

where $y_{j}$ is a smoothed data point corresponding to observed value y_j_. We can observe, by studying the data from a day of processing of a batch of product, that the operator has left the fluid bed dryer preheating for more than 100 min (*x*-axis), at which point the curve begins to descend. At this point, the next phase commences where the granulated product is loaded into the fluid bed dryer to begin drying. It can also be observed that the maximum difference between the air inlet and outlet temperatures in both cases is between 12 and 15 degrees (*y*-axis). So, it can be deduced that hot air is being introduced into the fluid bed dryer for a longer period than necessary (since the temperature differences between the inlet and outlet air remain stable). Thus, the operator is lengthening the process longer than the necessary time, during which energy is being consumed and wasted.

### 6.2. Verification of the Hypothesis with Historical Data from Production Batches

**Figure 9:**
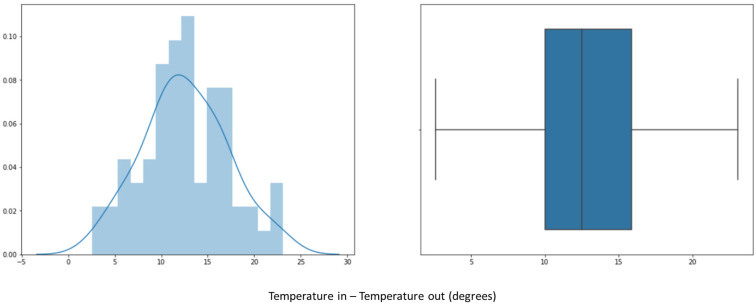
Temperature distribution during preheating for 200 batches of product: histogram (**left**); box plot (**right**).

Once key trends have been identified in selected sample days (Section 5), the next step is to evaluate the preheating phase of all the 200 product batches available in the 700,000 signals (about two years of data). We can see in Figure 9 how the inlet (TAE) and outlet (TAS) temperature difference is distributed. We observe that the median is around 12 degrees, which is similar to the median of the previous sample containing two batches (Figure 8).

**Figure 10:**
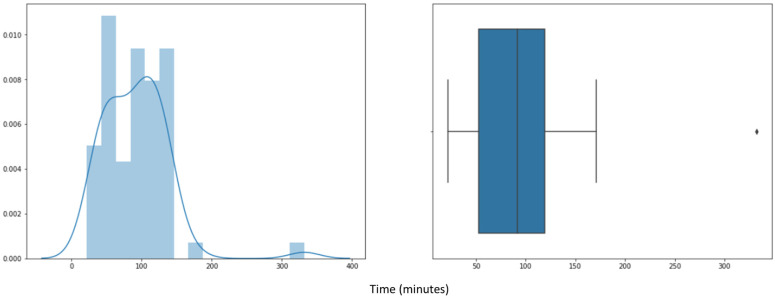
Distribution of preheating completion times for 200 product batches: histogram (**left**); box plot (**right**).

In addition, in Figure 10, we can observe the duration in minutes of the preheating phase for all the batches analyzed. Figure 10 shows that the preheating duration varies mainly between 45 and 115 min, with the median being around 75 min.

This time variability depends on when the operator has started and finished the heating process. Since it is a manual process due to the age of the fluid bed dryer (more than 20 years), there are operators who keep the fluid bed dryer preheating for about 45 min, whereas other operators keep the fluid bed dryer preheating for up to 115 min. Although there is no product in the preheating stage, the preheating conditions are different depending on the product to be processed later, since not all products dry at the same temperature. This causes the differential in temperature to not always be the same. Additionally, we have lots that are the first batches of a given product (first series), and others are not. The first series will probably show a longer time to reach the same point than those that follow. It is this variability that makes it important to have a system which has an optimization capability.

Therefore, a “look-up table” could be defined, followed by a data model that relates the pre-heating time with the independent variables mentioned, that is, the batch/product, batch order in the series, etc. The operator would enter the independent variables into the control system, and the control system would calculate and apply the corresponding optimal time.

**Figure 11:**
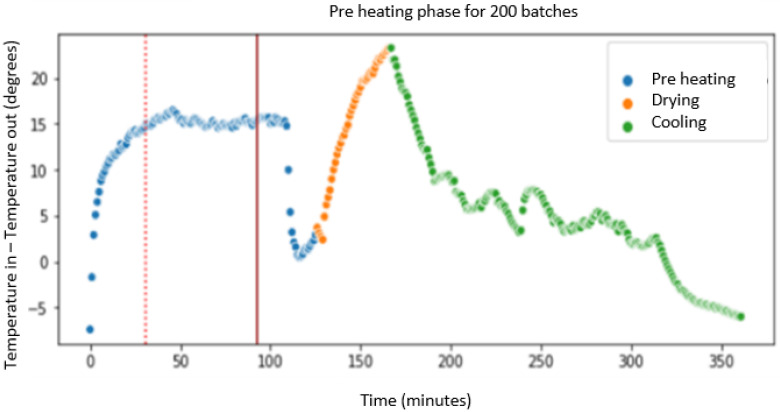
Average of the measurements of the 200 batches, time vs. temperature. Potential time savings in the preheating process shown by vertical lines.

As a conclusion drawn from the pre-processing and scanning of the signals, illustrated in Figure 7, Figure 8, Figure 9 and Figure 10, it can be affirmed that the preheating phase lasts longer than necessary. Once the 12.5° temperature difference between the inlet and outlet air is reached, the fluid bed dryer is at the correct temperature to proceed with the drying of the product. Therefore, from this point on, it is not necessary to continue heating. Next, we can see in Figure 11 how when applying the pre-processing and exploration techniques that have been commented previously, the complete process is visualized, including the preheating phase of the fluid bed dryer IF = 1 (blue dots 1.0), the drying phase of the product IF = 2 (orange points 2.0), and the cooling phase IF = 3 (green points 3.0). In Figure 11, the y-axis indicates the difference between inlet air temperature (TAE) and outlet (TAS), and the x-axis represents the elapsed time in minutes. Thus, as can be seen in Figure 8, the pre-heating phase temperature difference stabilizes between 12.5° and 15° (blue dots). This is the desired stable state required as a precondition for the drying phase to commence. Therefore, further pre-heating (as was done in the previous system which had a fixed time period) is unnecessary, and the system can (dynamically) commence the drying phase once this state is detected, thus saving time and energy consumption.

The time in minutes (*x*-axis) between the red vertical lines corresponds to the average time wasted (approx. more than 50 min that the fluid bed dryer is preheating unnecessarily) since the fluid bed dryer is already at its optimum point (approximate difference of 12.5 degrees between the inlet and outlet temperatures) to be able to start drying the product (phase 2 with dots in orange). This potential saving in time and energy consumption also implies a reduction in the entire process (which lasts about 350 min: from the start time of fluid bed dryer preheating until end of cooling). This amounts to a saving of approximately 50–60 min per batch and the corresponding energy consumption reduction. The power of the fan’s fluid bed dryer used is 18.5 kWh, so if we consider that we can reduce, on average, almost an hour of preheating time where the fan is working at full power (sending hot air at more than 45 degrees with a flow of 2000 m^3^), we calculate an energy saving for each batch of 18.5 kWh. Remember that the fluid bed dryer processes approximately 200 batches per year, giving an estimated annual energy saving of approximately 3700 kWh (200 batches/year × 18.5 kWh/batch).

## 7. Conclusions and Future Work

This paper has provided a practical example of how to use an EDA (Exploratory Data Analysis) methodology to analyze and optimize a large-scale drug production process, such as the drying process of solid drugs (pharmaceutical granules) through a fluid bed dryer. The problem of how to optimize energy consumption in fluid bed dryers for pharmaceutical granules has been addressed, obtaining average reduction results of almost half the time used by the fluid bed dryer in the process (average 1 h per batch), and a considerable saving in energy consumption (18.5 kWh) per batch. This has been achieved by using advanced analytical data exploration and pre-processing techniques on more than 700,000 data records from 56 sensor signals captured with 1-min time intervals from a Fielder-Aeromatic MP 6/8 fluid bed dryer. As a next step, we propose connecting the fluid bed dryer sensors to a cloud computing platform in real time. Then, from the results of the data exploration, develop a data model using machine learning algorithms. This model will provide the operator with a prediction of the time required to complete the preheating process via the SCADA interface. As future research, the same technique could be used for the drying and cooling processes of the fluid bed dryer, as well as for the time-consuming process of cleaning and disinfection of the equipment once a batch is completed. This methodology and technique can also be applied to other types of machines within the industrial drug production process, such as compactors, coaters, mixers, etc.

## Figures and Tables

**Table 1:** Example of signals used for the experiment.

	Power Impeller [Kw]	Air Flow [m³/h]	Inlet Air Temperature [°C]	Outlet Air Temperature [°C]
count	1441.000000	1441.000000	1441.000000	1441.000000
mean	0.208952	1040.490632	23.295212	27.635045
std	0.944463	1366.454053	6.842901	7.364493
min	0.000000	−58.000000	10.700000	22.100000
25%	0.000000	−55.000000	16.800000	22.900000
50%	0.000000	−55.000000	27.700000	23.400000
75%	0.100000	2471.000000	29.500000	30.800000
max	11.600000	4042.000000	31.600000	47.900000

**Table 2:** Signals used for the experiment.

Abbreviation	Sensor	Average	Max	Min
IF	Phase Indicator (1, 2, 3)	N/A	3	1
TAE	Temperature inlet Air	23.2	52.8	0
TAS	Temperature outlet Air	27.6	47.9	0
CAE	Inlet Air Flow	1040	4042	0
MOT	Fan Motor	19.8	151	0

## Data Availability

Dataset not available due to privacy as it belongs to a pharmaceutical company located in Barcelona, Spain.
